# AI-Enhanced 3D Models in Global Virtual Reality Case Conferences for Surgical Care in a Low-Income Country: Exploratory Study

**DOI:** 10.2196/69300

**Published:** 2025-08-18

**Authors:** Miriam Obst, Jan Arensmeyer, Henrik Bonsmann, Andreas Kolbinger, Joel Kigenyi, Francis Oneka, Benard Owere, Joachim Schmidt, Philipp Feodorovici, Jan Wynands

**Affiliations:** 1 Faculty of Medicine, University of Bonn, Bonn, Germany; 2 Bonn Surgical Technology Centre (BOSTER), University Hospital Bonn, Joseph-Schumpert-Allee 1, Bonn, 53227, Germany, +491752721843; 3 Division of Thoracic Surgery, Department of Surgery, University Hospital Bonn, Bonn, Germany; 4 Medical Practice of Reconstructive and Aesthetic Surgery Pfaffenhofen, Pfaffenhofen, Germany; 5 Lamu Medical Centre for Reconstructive and Global Surgery, Jinja, Uganda; 6 Department of Thoracic Surgery, Helios Hsopital Bonn/Rhein-Sieg, Bonn, Germany; 7 Section Global Health, Institute of Hygiene and Public Health, University Hospital Bonn, Bonn, Germany

**Keywords:** 3D scanning, artificial intelligence, virtual reality, extended reality, metaverse, spatial computing, global surgery, reconstructive surgery

## Abstract

**Background:**

Approximately 5 billion people worldwide lack adequate access to surgical care, primarily in the Global South. Especially in crisis regions and war zones, telemedical applications may enhance health services. This study explores the feasibility of using artificial intelligence (AI)-enhanced 3D imaging and extended reality (XR) technologies for intercontinental surgical case conferences in a low-resource scenario in Uganda. Our pilot study aims to assess the value of these technologies to address the lack of surgical resources and multilateral knowledge exchange.

**Objective:**

This study intends to determine the feasibility of using new AI-enhanced image modeling technology within an immersive spatial XR scenario to collaboratively and remotely assess reconstructive patient cases in the resource-limited country of Uganda.

**Methods:**

Within a surgical camp at Lamu Medical Centre, Uganda, 3D models of patients’ conditions were created using a smartphone app. Digital models were generated from photographs taken on-site and processed into 3D formats to be visualized in virtual case conferences. Here, surgeons from Uganda and Germany used virtual reality (VR) headsets to collaboratively discuss case strategies while marking surgical approaches on each digital patient model.

**Results:**

The study included 15 patients requiring reconstructive surgery, with a diverse range of conditions. The use of XR technology facilitated detailed visualization and discussion of surgical strategies. The process was time-efficient, with a total of under 8 minutes per case for data acquisition and model creation, and resource-efficient with surgeons reporting sufficient quality of smartphone-derived models. Valuable user experience and precise interaction during the VR case processing were found, underlining its potential to improve surgical planning and patient care in resource-limited settings.

**Conclusions:**

The findings indicate that AI-enhanced 3D imaging and immersive virtual communication platforms are valuable tools for integrative surgical case assessments. The cost-effectiveness of the used consumer solutions should be especially beneficial for low-resource environments. While the study demonstrates the feasibility of this approach, further research is needed to explore a broader application and impact of these technologies in global health. The study highlights the potential of XR to enhance training and surgical precision, contributing to better health care outcomes in underserved regions.

## Introduction

### Background

A modeling study has demonstrated that approximately 5 billion people worldwide lack adequate access to surgical care [1]. This issue particularly affects regions in the Global South, including Sub-Saharan Africa, South America, and Asia. To target this issue, “Global Surgery” has been initiated and prioritized in the past decade by the World Health Assembly as a concept to improve and equalize surgical care across international health systems [2]. The Lancet Commission of Global Surgery particularly emphasizes the role of innovation and mobile health to address the accessibility of surgical expertise [3]. Rural surgeons in low-income countries, who must provide broad surgical skills, play a critical role in advancing Global Surgery [4]. In crisis regions and war zones, remote medical assistance, such as telemedical consulting, can enhance health service delivery, as currently demonstrated in the Gaza Strip [5].

Limited funding for medical technology is a key burden holding back the evolution of treatment practice as well as medical education in low-income countries [6]. In high-income countries, an increasing variety of high-end technologies in health care systems is available, whereas limited resources in low-income countries could especially necessitate frugal designs [7]. Uganda is an East African country with a population of 45.5 million people [8]. The World Bank classifies Uganda as a low-income country per its income classification [9]. The country has a severe shortage of qualified surgeons, with fewer than one per 100,000 people, exacerbating challenges such as geographic isolation, low health literacy, limited financial resources, and insufficient health care infrastructure [10-13]. In many urgent cases, for example, in frequent road traffic accidents, acid attacks, or fire burns, patients cannot receive the necessary immediate treatment. Individuals surviving the initial trauma later often become affected by preventable contractures and immobility, eventually necessitating plastic and reconstructive surgery [14]. A recent review analyzes the Ugandan health care system and emphasizes the need for investments and enhancements in both new and existing infrastructure as well as in the further education of health care workers [15].

In reconstructive surgery, a 3D understanding of individual pathologies located in surface anatomy is pivotal. This applies to treatment approaches for both traumatically induced injuries, such as burns, accident- or war-related wounds, as well as benign space-occupying lesions or oncological entities.

Modern software for creating 3D models from 2D images, such as photogrammetry, has made significant strides in recent years. This technique analyzes multiple 2D images taken from various angles to generate accurate 3D models. AI-driven 3D reconstruction technologies further improve model creation, enhancing both speed and quality [16,17]. These technologies allow high-quality 3D model creation even from frugal sources like smartphone cameras.

The application of extended reality (XR), which includes virtual reality (VR), augmented reality (AR), and mixed reality (MR), is expanding rapidly in the medical field [18,19]. However, access to these technologies is not equally distributed from a global perspective. In surgery, the technology allows preoperative planning using patient-specific imaging in 3D environments and the training of surgical procedures through immersive simulations [20-22]. Where available, XR has a positive impact on surgical training [23-25]. Computed tomographic (CT) and magnetic resonance imaging (MRI) scans can be reconstructed into a 3D image and displayed with the use of head-mounted displays (HMDs), commonly known as VR headsets. Novel explorations even demonstrate intraoperative holographic overlays of 3D reconstructed images [26]. For the user, visualizing the patient data in a 3D stereoscopic view reduces the cognitive work needed to assess the 3D information out of 2D scan slices and similar imaging technologies [27-29]. The use of 3D-reconstructed medical imaging has been analyzed in several studies, often in the context of addressing challenges in surgical strategies for complex oncological scenarios [26,30-32].

Therefore, in lower-income regions without access to radiological imaging, photogrammetric modeling could be an additional tool for patient data acquisition. Besides novel possibilities of 3D image representation, the assembly of health care professionals in immersive virtual spaces from different locations opens new approaches in future medical conferencing [33].

The employment of XR and AI-supported smartphone apps facilitates surface visualization in a manner that is uncomplicated, expeditious, cost-effective, and radiation-free, thereby offering a distinct advantage over radiological imaging methods. Our study emphasizes frugal and robust technological solutions by using widely available, consumer-grade hardware that is well-suited to low-resource and rural settings, where access to surgical infrastructure and education remains limited. By validating the feasibility, we propose a potential pathway for the broader adoption of surgical training and assistance tools, ultimately contributing to more equitable access to surgical care worldwide.

No original empirical data or scientific work that shows the integration of XR technology with AI-enhanced image reconstruction for the purpose of global collaborative surgical planning in the Global South has been identified. Thus, an explorative study design was carried out to provide firsthand and original data [34]. To the best of our knowledge, we describe a pioneering explorative study that uses collaborative XR for case conferencing and assessment using AI-enhanced 3D surface models of patients from rural Uganda in reconstructive surgery while executed only on affordable consumer-based hardware platforms.

### Objective

This study intends to determine the feasibility of using new AI-enhanced image modeling technology within an immersive spatial XR scenario to collaboratively and remotely assess reconstructive patient cases in the resource-limited country of Uganda.

## Methods

### Study Cohort

Surgeons participating in the surgical camp by INTERPLAST-Germany e.V. at Lamu Medical Centre for Reconstructive and Global Surgery (Lamu) in Jinja, Uganda (October 22 until November 4, 2023) were invited to be involved in this exploratory study. Lamu is a private medical facility that regularly hosts international surgical camps. During this period, patients with a variety of potential reconstructive surgical conditions were included based on the following criteria.

Inclusion criteria:

- Having a condition that requires evaluation for reconstructive surgery (eg, fire or acid burns, amputations, scars, or skin and soft tissue masses or tumors).
- Being over 18 years of age, legally competent, and having provided written informed consent.

Exclusion criteria:

- Having an underlying condition that does not allow for anesthesia or surgery.
- Testing positive for COVID-19 or exhibiting symptoms of a COVID-19 infection.

Patient eligibility was assessed through a comprehensive anamnesis and physical examination relevant to the presenting pathology. When available, medical records were also reviewed.

### Workflow

**Figure 1.**
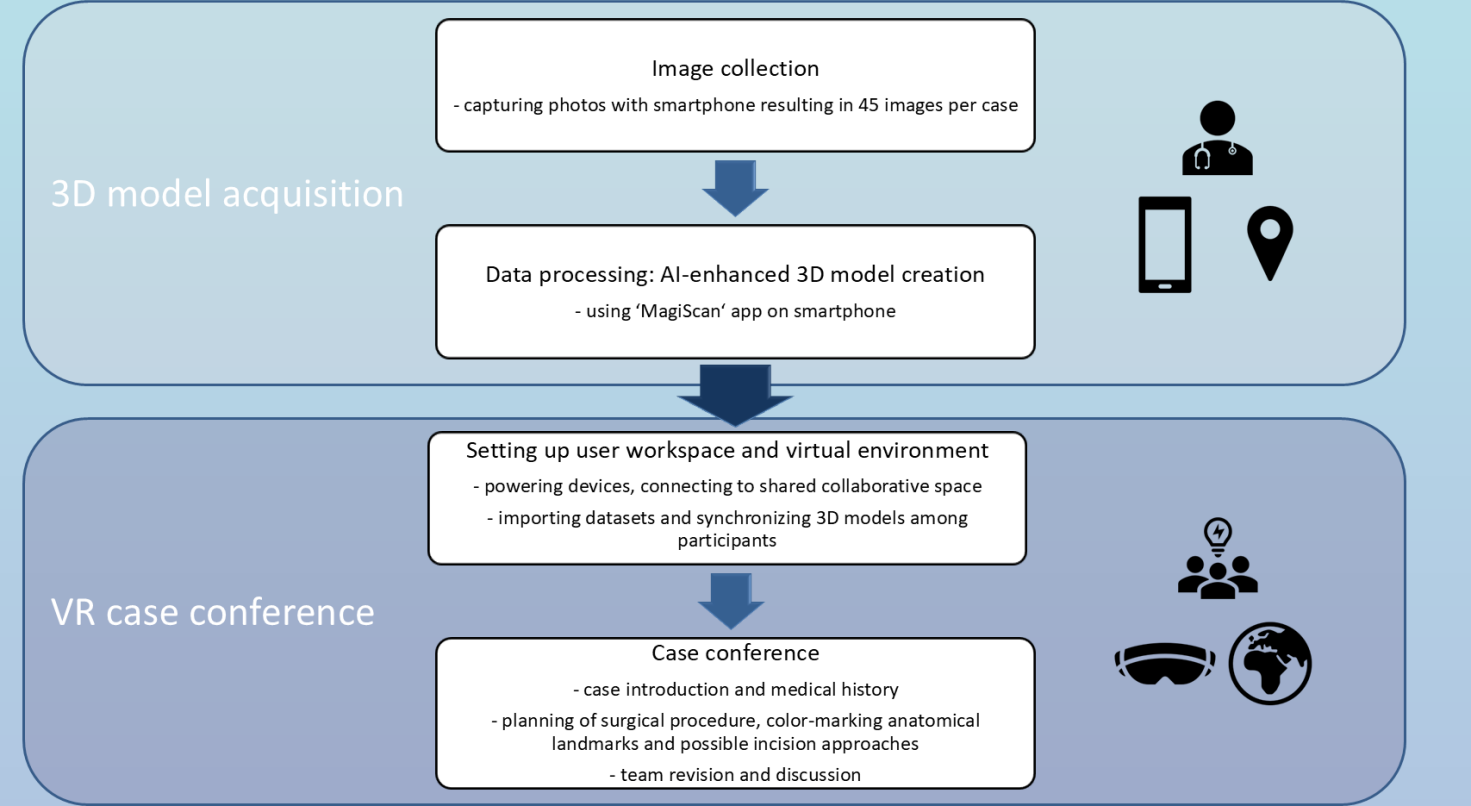
The workflow of this study encompasses 2 major phases.

After participants’ enrollment, data acquisition and processing were conducted. Newly created datasets were prepared on-site while VR case conferences were scheduled. Figure 1 illustrates the study’s workflow, which encompasses 2 phases: the 3D model acquisition and the virtual reality case conference. Initially, the images were collected and then processed into a 3D reconstructed model using an artificial intelligence-enhanced smartphone app. Following the successful setup, the virtual reality case conference was conducted, during which each patient’s individual pathology and surgical strategy, including the marking of potential incision lines and flap approaches, were discussed.

### Creation of 3D Model

The 3D models of the participants’ conditions were created using the AI-enhanced smartphone app MagiScan (App version 1.8), developed by MagiScan Inc. A Google Pixel 7a smartphone, Google Inc., equipped with the Android operating system (version 13), was used for scanning. Each patient underwent a photographic session. A total of 45 images were taken from various angles and orientations as the photographer executed a 360° circumferential movement around the patient. The patient was instructed to maintain a stable position, either standing or sitting, to ensure a comprehensive visualization of the pathology from all relevant angles. Inadequate lighting conditions are the most common cause of artifacts, such as inaccurate surface discontinuities. It is essential to avoid spotlights and instead use indirect lighting to ensure optimal accuracy. These photos were subsequently uploaded into the app and autonomously processed on a dedicated secure storage in a German-based data center (Frankfurt, Germany), and maintain guidelines of the EU General Data Protection Regulation (GDPR). The patient data was anonymized by assigning a numeric pseudonym.

Raw photo data were transformed into 3D models, the resulting digital representation of the patient was exported in Graphics Library Transmission Format (GLTF) to be further imported into the VR platform.

### VR Hardware and Software

Two different types of VR headsets (HMDs) were used to mitigate the risk of insufficient functionality in one of the headsets and to ensure flexibility in setup by including both wired and wireless options. The Meta Quest 2 operated wirelessly as a standalone device, whereas the Oculus Rift S was wired to a workstation. Manual interaction was facilitated through VR controllers wirelessly connected to the HMDs. Both headsets enabled a functional setup. The headsets provide a similar technical performance and user experience.

The used software was XR Dissection Master (Version V0.14.3) by Medicalholodeck, installed as a standalone device.

### Virtual Case Conference

The virtual case conference initiated with a visual and audio check-up. In each setting, a Ugandan surgeon from Lamu (n=2) and a remotely integrated surgeon affiliated with INTERPLAST-Germany e.V. (n=2) were equipped with a set of VR glasses and controllers, allowing them to participate in the shared digital environment. All sessions were accompanied by the research assistant and principal investigator, who ensured sufficient setup and data acquisition. The 3D datasets were uploaded onto the XR platform and automatically synchronized among participants. Case discussions commenced with an introduction of the participating surgeons. Thereafter, the patient’s medical history and ongoing diagnosis were presented by the on-site surgeon. Possible procedures and tailored surgical approaches for each case were discussed. Throughout the case discussion, the app allowed users to zoom in and out of the 3D model, as well as rotate and move it as required. A toolbar provided a pen with various colors and stroke widths for highlighting and annotating specific areas of the object. During the interaction, the surgeons were represented by individually colored avatars, including virtual hands. After the mutual exchange, an individual treatment plan was proposed, and a final team revision was conducted for each case.

The case conferences were recorded on a server based at the University Hospital Bonn to later assess the durations of conference time sequences. The surgeons’ feedback was collected through semistructured interviews, including Likert scale items on user experience and content quality.

### Ethical Considerations

The study was conducted in accordance with the Declaration of Helsinki and received ethical approval from the institutional review board of the Mildmay Uganda Research Ethics Committee (approved September 20, 2023; Ref. MUREC-2023‐299) and the ethics committee of the University of Bonn (approved August 15, 2023; Ref. 235/23-EP). Administrative clearance was granted by the Ugandan Ministry of Health on October 11, 2023. The study was registered with the Ugandan Council for Science and Technology (UNCST), which issued a research permit on October 23, 2023 (Ref. HS3198ES). Written informed consent was obtained from all participating patients and surgeons after they received comprehensive written and verbal information about the study. The participants were assured their right to withdraw from this study at any time. To safeguard anonymity and prevent any possibility of direct identification, a numerical pseudonym was assigned. Data access was limited exclusively to authorized members of the research team. No compensation was given to the participants.

## Results

A total of 15 patients from the surgical camp conducted by INTERPLAST-Germany e.V. were included in this study (n=15). Four categories of working diagnosis were established according to the patient’s surgical condition. Each patient’s medical history and surgical procedure were discussed in a total of 3 intercontinental virtual case conferences. The meeting within an immersive virtual room facilitated real-time communication and interactive engagement with the 3D models of the patients. A recording of the virtual case conference is available as Multimedia Appendix 1.

The cohort consisted of 5 patients who endured burn contractures resulting from fire, hot water, or acid. Seven patients exhibited a variety of surface tumors, including lipoma, ganglion, cysts, or unknown lesions of the soft tissue. Two patients presented with chronic wounds. One patient showed soft tissue damage following trauma. The entities appeared in different locations that have been arranged into 3 distinct categories: Limb (8 patients), Head and Neck (7 patients), Trunk (1 patient). The summarized data of the cohort are presented in Table 1.

The median time for virtual case conferences was 3 minutes and 1 second, with a range from 1 minute and 33 seconds to 8 minutes and 14 seconds. Case introduction and medical history took a median of 50 seconds, while planning the surgical procedure had a median time of 1 minute and 20 seconds. Discussion and team revision took a median of 44 seconds. All time metrics related to data acquisition and 3D model creation are summarized in Table 2. The time metrics for the virtual case conferences are presented in Table 3. In some instances, team revision was not conducted if no additional questions or ambiguity arose following the discussion of surgical approaches and interacting with the 3D-reconstructed model.

Two general surgeons from Uganda and 2 reconstructive surgeons from Germany, affiliated with Interplast, conducted the XR case evaluations. During the virtual intercontinental case conferences, the surgeons actively engaged the app’s tools to outline anatomical landmarks and potential incision lines. Possible flap mobilizations and the placement of skin grafts were collaboratively marked on the patient models within the immersive setting to explore potential surgical strategies. The final surgical techniques included a jumping man flap, a supraclavicular flap, and a latissimus dorsi flap. All approaches were outlined and discussed.

**Table 1.** Overview of patient cohort.

Variable	Value
Overall group (count), N (%)	15 (100)
Sex, n (%)	
Female	5 (33.33)
Male	10 (66.66)
Age (years)	
Mean (SD)	40.1(15.88)
Range (years)	18‐75
Working diagnosis, n (%)	
Burn (fire, acid, hot water)	5 (33)
Trauma with soft tissue injury	1 (7)
Soft tissue space occupation or tumor	7 (47)
Chronic wound	2 (13)
Location, n (%)	
Head and neck	6 (40)
Trunk	1 (7)
Limb	8 (53)

**Table 2.** Metrics for 3D model reconstruction.

Data acquisition time (n=7)	Time (min: sec)
Photographic session	
Median time per case	02:33
Range	01:56 – 02:56
Processing data into 3D-reconstructed model	
Median time per case	03:10
Range	02:12 – 04:14
Total time per case	
Median	05:43
Range	05:08 – 07:13

**Table 3.** Metrics for virtual case conference.

Virtual case conference time sequences (n=15)	Time (min: sec)
Case introduction and medical history	
Median time per case	00:50
Range	00:30 – 02:11
Planning of surgical procedure	
Median time per case	01:20
Range	00:24 – 06:40
Discussion and team revision	
Median time per case	00:44
Range	00:00 – 01:13
Total virtual case conference	
Median time per case	03:01
Range	01:33 – 08:14

**Figure 2.**
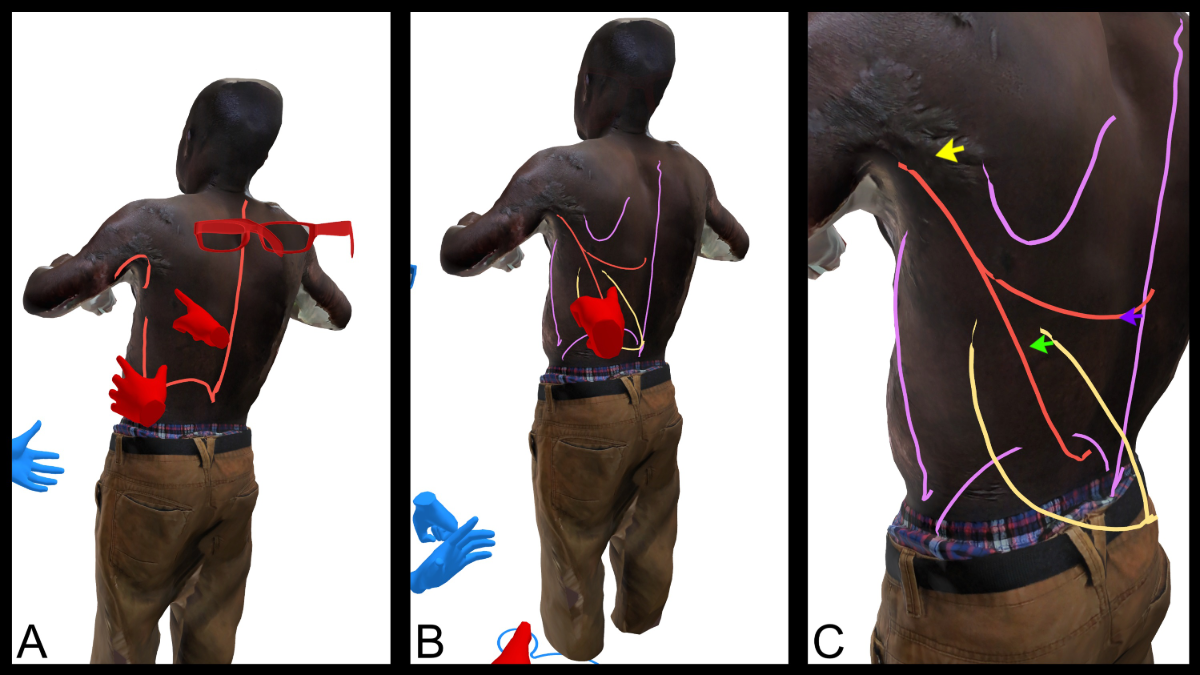
Virtual reality images of a patient with postburn contractures of both axillae. 3D model showing the patient’s back with postburn contracture of the left axilla, marking the borders of the latissimus dorsi muscle in red color. (**A**) Placing anatomical landmarks (inferior scapula, iliac crest, mid-axillary line, and dorsal median line) in purple color, drawing the muscle’s main vessel and its branches (thoracodorsal artery) as well as its origin (subscapular artery) in red color, marking the skin paddle to be integrated in the flap (musculocutaneous flap) in yellow color. (**B**) Enlarged picture with landmarks and skin paddle, focusing on the vessel axis (red color), giving particular attention to its origin (yellow arrow) and the course of branches (green and blue arrows). (**C**) The enlarged picture allows a clearer imaging of the skin texture (eg, scar tissue close to the vascular origin); Images A and B show the participating surgeons as avatars; red hands and glasses: presenting surgeon, blue hands: attending surgeon.

Figure 2 demonstrates a VR image of a patient with postburn contractures. Major anatomical landmarks are highlighted on the 3D model in Figure 2A, vascular structures are marked in Figure 2B, and key vessel axes along with their origins are shown in Figure 2C.

**Figure 3.**
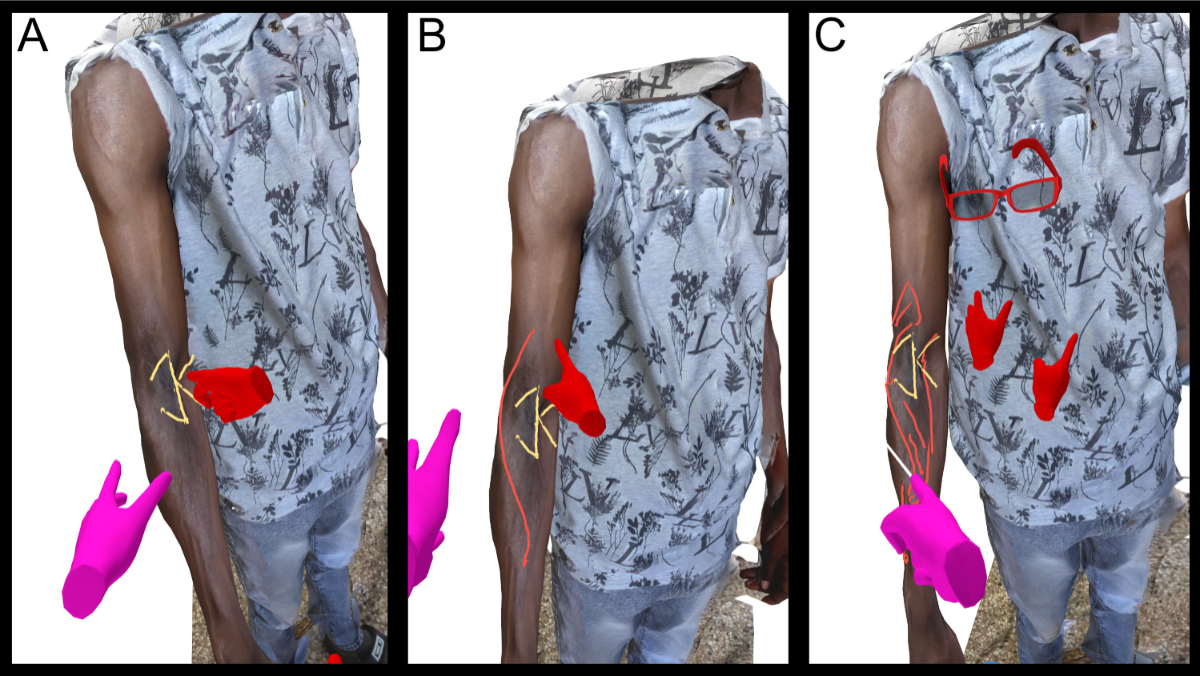
Point-of-View virtual reality images of a patient with postburn contractures of the right elbow. (A) 3D model showing the patient’s ventral aspect of the right elbow with the planned incision line and local “jumping man” flap design marked in yellow. (B) Extensive scarring in the area of the cubital groove, as well as the neighboring tissue in the latero-ventral area of the distal upper and proximal lower arm, is marked in red. (C) Detailed surface analysis and discussion of the adjacent scar tissue (shaded area in red) by attending surgeon (purple hands) and presenting surgeon (red hands and glasses).

Figure 3A presents a VR image of another patient with postburn contractures. The "jumping man flap," proposed as a therapeutic option, is marked in Figure 3B, and further areas of tissue mobilization are highlighted in Figure 3C.

**Figure 4.**
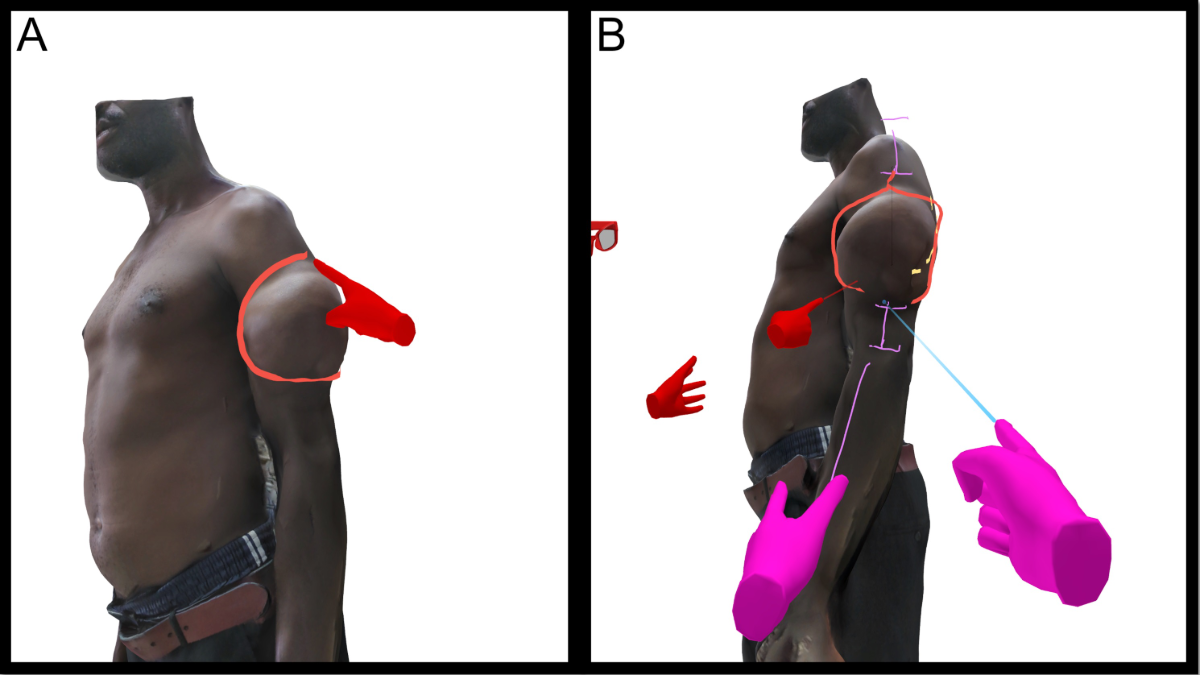
Patient model presenting with a painful lipoma of the lateral aspect of the left upper arm. (**A**) Outlined boundaries of the lipoma in red for an improved understanding of volume and dimension. (**B**) Marked preferred posterior incision line in yellow (dotted line) and anatomical landmarks (central axis) in purple color. Discussion and surgical planning by attending surgeon (purple hands) and presenting surgeon (red hands and glasses).

Figure 4A depicts a patient with a large lipoma, along with the proposed incision line for surgical treatment Figure 4B.

The objective of the surgeons’ evaluation was to assess their detailed perception of the 3D-reconstructed patient models and their integration into a VR-based case conference. Specifically, the evaluation focused on the quality and visibility of the 3D-reconstructed models, communication features, and overall feasibility. Surgeons’ feedback was collected through Likert-scale items. The frequency distribution of the response options was analyzed to compare the responses. Likert-scale results of the surgeon’s feedback demonstrated that all 4 participating surgeons (4/4, 100%) strongly agreed that the 3D reconstructed models were of high quality. Regarding the visualization of large entities, all surgeons (4/4, 100%) found it satisfactory, while 2 surgeons (2/4, 50%) expressed satisfaction with the visibility of smaller entities, such as ganglions and finger contractures. All surgeons (4/4, 100%) agreed that the audio and visual communication tools were sufficient. No surgeons (0/4, 0%) reported any significant motion sickness, with only 1 surgeon reporting to have experienced slight motion sickness. However, at no point did this lead to the termination of the VR session or the need for a pause. Furthermore, all surgeons (4/4, 100%) affirmed that the technology could enhance surgical care in low-resource settings and could be integrated into routine practice for reconstructive surgery case discussions. The detailed results are provided in Multimedia Appendix 2.

## Discussion

### Principal Results

Our findings demonstrated a novel, intercontinental immersive surgical health care approach. We initiated and explored a solution for remote case conferencing and interactive assessments of surgical strategies on digital patient models, providing insights into the requirements and effectiveness of AI-enhanced modeling and XR setups.

The VR-based case assessment process was time efficient, as reflected in the median metrics, enabling multiple surgeons to conduct remote, simultaneous, and detailed evaluations without being physically colocated. This collaborative setup supports improved resource allocation. Various surgical entities and conditions were assessed, with surgeons outlining individualized strategies for each case. The dynamic structure of the case conferences led to variations in timing across different assessment phases.

The accuracy of 3D reconstruction and representation of smaller lesions, such as small lipomas and ganglions, was limited, whereas larger entities were depicted with great accuracy. The smartphone-based 3D reconstructions were acquired in a reasonable timeframe but occasionally showed minor artifacts. Compared with conventional photogrammetry, AI-enhanced 3D reconstruction methods require significantly less time and computational resources [35], making them particularly valuable for rapid and cost-efficient 3D modeling in clinical settings. Ongoing developments in consumer mobile devices, particularly integrated light detection and ranging (LIDAR) technology, show promise for further applications and exploration in surgical practice, offering highly accurate 3D scanning capabilities that are already being used in the medical field [36].

### Immersive Communication and Surgical Collaboration

Intercontinental communication in the immersive environment was effective during the real-time conferences in both visual gestures and speech. After their initial involvement in virtual case conferences, all participating surgeons recognized the high potential of 3D representation in XR to improve surgical care in the Global South. This approach may even serve as a future standard of care in remote planning of reconstructive operations. This underlines the quality of the patient models as well as the effectiveness of the VR communication platform.

Although no directly comparable studies currently exist, related literature underscores the influence of XR in surgery and digital communication. Numerous studies have demonstrated the potential benefits of XR in surgical planning, navigation, intraoperative guidance, and training [19,37]. In addition, the impact of digital communication solutions on case preparation and collaboration further emphasizes the potential of these technologies in surgical practice [38].

### Limitations

This study aims to gain initial insights into the value and usability of the technology by implementing it during a temporal surgical camp to establish a standardized protocol. Consequently, the sample selection and composition may limit the representativeness of the patient sample compared to broader populations in public hospitals.

In addition, the small study population of this exploratory proof of principle limits the ability to draw meaningful conclusions about the technology’s impact on specific surgical pathologies or subentities. Furthermore, due to this design, the number of participating surgeons was limited, leaving room for a potential influence of individual bias.

### Opportunities of XR for Surgical Care in Low-Income Regions

This study confirms the feasibility of XR-based case assessments for reconstructive surgery in a low-income country using frugal, off-the-shelf hardware. Multidirectional, immersive, and interactive communication between rural surgeons, specialists, and the broader community in the field of reconstructive surgery can reduce barriers to effective case discussions, facilitating global knowledge exchange based on real patient data [39,40]. Our study has shown that these goals are achievable with frugal equipment and limited training, with hardware costs kept below 1000 US$, making the setup accessible and scalable [41]. However, consistent, low-latency internet connectivity is essential for effective real-time VR use. XR has already proven valuable in surgical education, planning, increased precision, reduced operative times, and optimized outcomes while minimizing resource demands [42]. XR presents a significant opportunity to further educate health care professionals in anatomy and surgical skills, especially in resource-limited settings where extensive training on cadavers or live models is often unavailable. Particularly, in plastic and reconstructive surgery, XR training solutions can play a valuable role [43].

### Conclusion and Outlook

Despite constrained access to modern medical technologies, this study demonstrates, for the first time, the feasibility of AI-enhanced 3D object reconstruction in conjunction with XR for reconstructive surgical assessment in a low-income country. Implementing such technologies in resource-poor settings across the Global South could significantly improve global surgery and reduce barriers to access to surgical care. It is suggested that medical AI applications need to be implemented equitably and inclusively from a global perspective [44].

While the affordability of VR headsets and AI-enabled mobile scanning continues to improve, financial barriers and infrastructural limitations, particularly internet access and initial investment, remain critical challenges [7]. Nonetheless, facilitating access to these technologies for rural health care providers could significantly expand their impact and adoption.

To scale this approach, larger prospective studies are needed to evaluate its effects on patient outcomes, cost- and time-effectiveness, and treatment workflows. Priorities for future research include exploring the implications of immersive planning on surgical decision-making, patient access, and rural outreach as patients often must travel long distances. As emphasized by the Lancet Commission, international collaboration and global visibility are essential to advance this solution beyond its pilot stage and realize its full potential in global surgery.

## Supplementary material

10.2196/69300 Multimedia Appendix 1 Recording of the virtual case conference.

10.2196/69300 Multimedia Appendix 2 Likert scale evaluation.

## Abbreviations

AI: artificial intelligence
AR: augmented reality
CT: computed tomographic
GDPR: General Data Protection Regulation
GLTF: Graphics Library Transmission Format
HMD: head-mounted displays
LIDAR: light detection and ranging
MR: mixed reality
MRI: magnetic resonance imaging
UNCST: Ugandan Council for Science and Technology
VR: virtual reality
XR: extended reality

## References

[1] Alkire BC, Raykar NP, Shrime MG (2015). Global access to surgical care: a modelling study. Lancet Glob Health.

[2] Price R, Makasa E, Hollands M (2015). World Health Assembly Resolution WHA68.15: “Strengthening Emergency and Essential Surgical Care and Anesthesia as a Component of Universal Health Coverage”—addressing the public health gaps arising from lack of safe, affordable and accessible surgical and anesthetic services. World J Surg.

[3] Meara JG, Leather AJM, Hagander L (2015). Global surgery 2030: evidence and solutions for achieving health, welfare, and economic development. Lancet.

[4] Kim EK, Dutta R, Roy N, Raykar N (2022). Rural surgery as global surgery before global surgery. BMJ Glob Health.

[5] Alser K, Mallah SI, El-Oun YRA (2024). Trauma care supported through a global telemedicine initiative during the 2023-24 military assault on the Gaza Strip, occupied Palestinian territory: a case series. Lancet.

[6] Clifford GD (2016). E-health in low to middle income countries. J Med Eng Technol.

[7] Howitt P, Darzi A, Yang GZ (2012). Technologies for global health. Lancet.

[8] (2023). Uganda Profile. Uganda Bureau of Statistics.

[9] (2022). World Bank Country Classifications by Income Level (Uganda). World Bank.

[10] Nwanna-Nzewunwa OC, Ajiko MM, Kirya F (2016). Barriers and facilitators of surgical care in rural Uganda: a mixed methods study. J Surg Res.

[11] Butler EK, Tran TM, Nagarajan N (2017). Epidemiology of pediatric surgical needs in low-income countries. PLOS ONE.

[12] Fuller AT, Corley J, Tran TM (2018). Prevalence of surgically untreated face, head, and neck conditions in Uganda: a cross-sectional nationwide household survey. World Neurosurg.

[13] Davé DR, Nagarjan N, Canner JK, Kushner AL, Wong GB, SOSAS4 Research Group (2020). Global burden of craniofacial disorders: where should volunteering plastic surgeons and governments focus their care?. J Craniofac Surg.

[14] Hodges S, Wilson J, Hodges A (2009). Plastic and reconstructive surgery in Uganda--10 years experience. Paediatr Anaesth.

[15] Turyamureba M, Yawe B, Oryema JB (2023). Health care delivery system in Uganda: a review. Tanzan J Health Res.

[16] Müller T, Evans A, Schied C, Keller A (2022). Instant neural graphics primitives with a multiresolution hash encoding. ACM Trans Graph.

[17] Miyake K (2024). Evaluating the reliability of three-dimensional models constructed photogrammetry software 3DF Zephyr by measuring joint angles of fingers: a comparison to a conventional goniometer. J Plast Reconstr Surg.

[18] Verhey JT, Haglin JM, Verhey EM, Hartigan DE (2020). Virtual, augmented, and mixed reality applications in orthopedic surgery. Int J Med Robot Comput Assist Surg.

[19] Zhang J, Lu V, Khanduja V (2023). The impact of extended reality on surgery: a scoping review. Int Orthop.

[20] Sun P, Zhao Y, Men J (2023). Application of virtual and augmented reality technology in hip surgery: systematic review. J Med Internet Res.

[21] Sayadi LR, Naides A, Eng M (2019). The new frontier: a review of augmented reality and virtual reality in plastic surgery. Aesthet Surg J.

[22] Yammine K, Violato C (2015). A meta-analysis of the educational effectiveness of three-dimensional visualization technologies in teaching anatomy. Anat Sci Educ.

[23] Mao RQ, Lan L, Kay J (2021). Immersive virtual reality for surgical training: a systematic review. J Surg Res.

[24] McKinney B, Dbeis A, Lamb A, Frousiakis P, Sweet S (2022). Virtual reality training in unicompartmental knee arthroplasty: a randomized, blinded trial. J Surg Educ.

[25] Sánchez-Margallo JA, Plaza de Miguel C, Fernández Anzules RA, Sánchez-Margallo FM (2021). Application of mixed reality in medical training and surgical planning focused on minimally invasive surgery. Front Virtual Real.

[26] Arensmeyer J, Bedetti B, Schnorr P (2024). A system for mixed-reality holographic overlays of real-time rendered 3D-reconstructed imaging using a video pass-through head-mounted display-a pathway to future navigation in chest wall surgery. J Clin Med.

[27] Yeo CT, MacDonald A, Ungi T (2018). Utility of 3D reconstruction of 2D liver computed tomography/magnetic resonance images as a surgical planning tool for residents in liver resection surgery. J Surg Educ.

[28] Wong KC, Sun EY, Wong IOL, Kumta SM (2023). Mixed reality improves 3D visualization and spatial awareness of bone tumors for surgical planning in orthopaedic oncology: a proof of concept study. Orthop Res Rev.

[29] Dey A, Chatburn A, Billinghurst M Exploration of an EEG-based cognitively adaptive training system in virtual reality.

[30] Feodorovici P, Schnorr P, Bedetti B, Zalepugas D, Schmidt J, Arensmeyer JC (2023). Collaborative virtual reality real-time 3D image editing for chest wall resections and reconstruction planning. Innovations (Phila).

[31] Thumerel M, Belaroussi Y, Prisciandaro E (2022). Immersive three-dimensional omputed tomography to plan chest wall resection for lung cancer. Ann Thorac Surg.

[32] Shirk JD, Thiel DD, Wallen EM (2019). Effect of 3-dimensional virtual reality models for surgical planning of robotic-assisted partial nephrectomy on surgical outcomes: a randomized clinical trial. JAMA Netw Open.

[33] Feodorovici P, Arensmeyer J, Schnorr P, Schmidt J (2023). [Extended Reality (XR) - Applications in Thoracic Surgery]. Zentralblatt fuer Chirurgie.

[34] Sandelowski M (2010). What’s in a name? Qualitative description revisited. Res Nurs Health.

[35] Kingsland K (2020). Comparative analysis of digital photogrammetry software for cultural heritage. Digit Appl Archaeol Cult Herit.

[36] Rudy HL, Wake N, Yee J, Garfein ES, Tepper OM (2020). Three-dimensional facial scanning at the fingertips of patients and surgeons: accuracy and precision testing of iPhone X three-dimensional scanner. Plast Reconstr Surg.

[37] Isikay I, Cekic E, Baylarov B, Tunc O, Hanalioglu S (2024). Narrative review of patient-specific 3D visualization and reality technologies in skull base neurosurgery: enhancements in surgical training, planning, and navigation. Front Surg.

[38] Hammer RD, Fowler D, Sheets LR, Siadimas A, Guo C, Prime MS (2020). Digital tumor board solutions have significant impact on case preparation. JCO Clin Cancer Inform.

[39] Diaka J, Van Damme W, Sere F, Benova L, van de Put W, Serneels S (2021). Leveraging smart glasses for telemedicine to improve primary healthcare services and referrals in a remote rural district, Kingandu, DRC, 2019-2020. Glob Health Action.

[40] Necker FN, Cholok DJ, Shaheen MS (2024). The reconstructive metaverse - collaboration in real-time shared mixed reality environments for microsurgical reconstruction. Surg Innov.

[41] Alsop T (2024). Comparison of virtual reality (VR) headsets worldwide in 2024, by price (in US dollars). Statista.

[42] Sullivan J, Skladman R, Varagur K (2024). From augmented to virtual reality in plastic surgery: blazing the trail to a new frontier. J Reconstr Microsurg.

[43] Bielsa VF (2021). Virtual reality simulation in plastic surgery training. Literature review. J Plast Reconstr Aesthet Surg.

[44] Liebrenz M, Bhugra D, Alibudbud R, Ventriglio A, Smith A (2024). AI in health care and the fragile pursuit of equity and social justice. Lancet.

