# Supplementary material for: AI-Enhanced 3D Models in Global Virtual Reality Case Conferences for Surgical Care in a Low-Income Country: Exploratory Study
Source: JMIR Form Res. 2025 Aug 18;9:e69300. doi: 10.2196/69300 (PMC12360718; doi:10.2196/69300)
Supplement: Supplementary file 1 [file formative-v9-e69300-s002.docx]

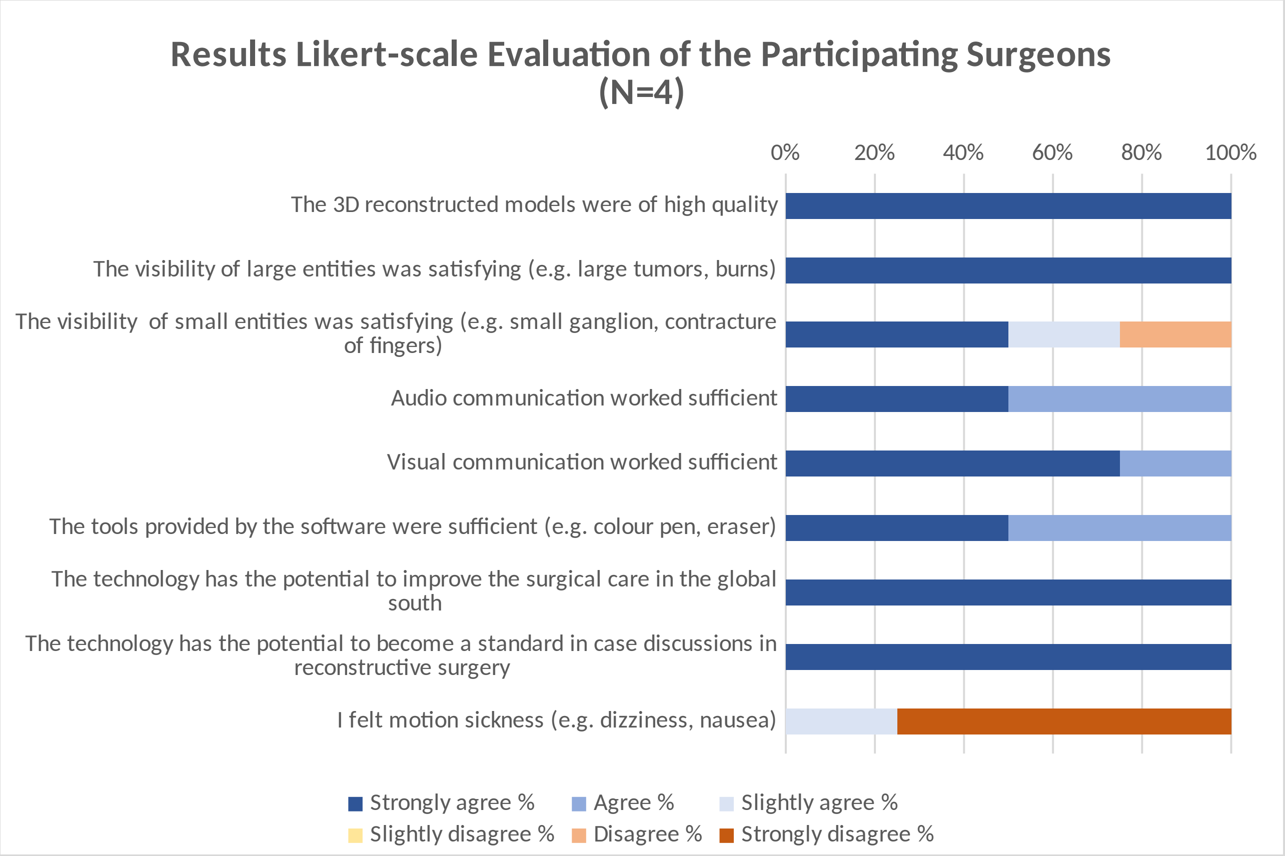

Results Likert-scale Evaluation of the Participating Surgeons (N=4)
0%
20%
40%
60%
80%
100%
The 3D reconstructed models were of high quality
The visibility of large entities was satisfying (e.g. large tumors, burns)
The visibility of small entities was satisfying (e.g. small ganglion, contracture of fingers)
Audio communication worked sufficient
Visual communication worked sufficient
The tools provided by the software were sufficient (e.g. colour pen, eraser)
The technology has the potential to improve the surgical care in the global south
The technology has the potential to become a standard in case discussions in reconstructive surgery
I felt motion sickness (e.g. dizziness, nausea)
Strongly agree %
Agree %
Slightly agree %
Slightly disagree %
Disagree %
Strongly disagree %
